# Allylsilanes in the synthesis of three to seven membered rings: the silylcuprate strategy

**DOI:** 10.1186/1860-5397-3-16

**Published:** 2007-05-22

**Authors:** Asunción Barbero, Francisco J Pulido, M Carmen Sañudo

**Affiliations:** 1 Departamento de Química Orgánica, Universidad de Valladolid, 47011 Valladolid, Spain

## Abstract

Addition of low order phenyldimethylsilylcyanocuprates to allenes followed by "*in situ*" reaction of the intermediate silylcuprate with electrophiles ("the silylcuprate strategy") provides new routes for the synthesis of functionalised allylsilanes, which undergo highly stereocontrolled silicon-assisted intramolecular cyclizations leading to three to seven membered ring-formation.

## Background

**Scheme 1:**
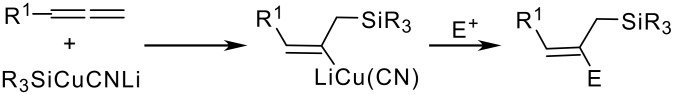
The silylcupration of allenes.

Organosilicon compounds and in particular allylsilanes have attracted considerable attention due to the increasing number of new methodologies that allow useful synthetic transformations. [1–2] Over the last decade allenes have emerged as one of the best sources for the synthesis of allylsilanes. [3] Although unactivated allenes do not easily undergo organometallic addition – and do not react with carbocuprates – they are readily attacked by metallocuprates. [4] In particular, simple allenes react with silylcuprates and stannylcuprates giving rise to a great variety of allyl- and vinylsilanes and stannanes with different substitution patterns. [5–6] The stoichiometry of the silylcuprate (higher or lower order) is responsible for the final regioselectivity of the reaction, leading selectively to allylsilanes when a lower order cyanosilylcuprate (R_3_SiCuCNLi) is used. [7] Moreover, the high reactivity of the intermediate allylsilane-vinylcuprate species toward electrophiles increases their synthetic potential (Scheme 1). [7–8]

A large number of electrophiles (alkyl and allyl halides, epoxides, ketones, α,β-unsaturated oxo compounds and acid chlorides, unsaturated nitriles and imines) have been successfully used in this reaction, leading to a wide range of functionalised allylsilanes, which are valuable intermediates for carbocyclic annulations. Effectively, the former substrates (containing a nucleophilic allylsilane unit and an electrophilic function) undergo "*intramolecular allylsilane terminated*" cyclizations when treated with Lewis acid, affording cyclic structures of different size.

In this account, we show a general survey of the recent advances in allylsilane chemistry and their significance as precursors for the synthesis of three to seven membered rings. We also highlight the contribution of our group to this field.

## Five and Six Membered Carbocycles

**Scheme 2:**
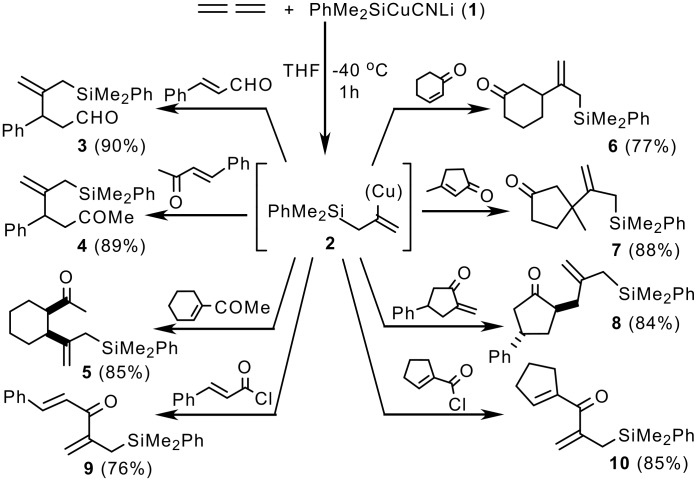
Silylcupration of 1,2-propadiene and reaction with oxo compounds.

Phenyldimethylsilylcyanocuprate **1**, prepared by mixing one equivalent of phenyldimethylsilyllithium and one equivalent of copper(I) cyanide, reacts with 1,2-propadiene (bubbled from lecture bottles) at -40°C leading to the intermediate copper species **2**, which on quenching with D_2_O undergoes deuterio-decupration introducing deuterium exclusively in the vinylic position C-2. As mentioned in the introduction, the use of lower order cuprates such as silylcyanocuprate **1** leads selectively to allylsilanes. Trapping of the intermediate vinylcuprate **2** with α,β-unsaturated oxocompounds provides an easy entry to the synthesis of oxoallylsilanes **3–8** which are useful synthons for cyclopentane annulations (Scheme 2). [7,9] Acid chlorides react with **2** affording divinyl ketones **9–10**.

**Scheme 3:**
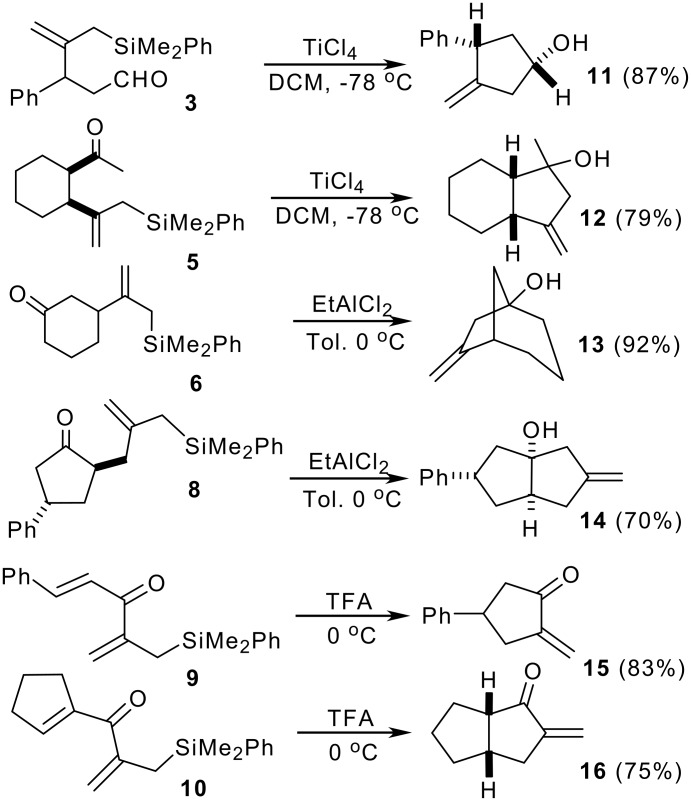
Silicon assisted cyclization of oxoallylsilanes.

Allylsilanes **3–8** carrying an electrophilic carbonyl moiety readily undergo intramolecular cyclization under Lewis acid catalysis. [10] Thus, silicon assisted cyclization of oxoallylsilanes **3–8** in the presence of TiCl_4_ or EtAlCl_2_ results in the formation of 3-methylene-1-cyclopentanols **11–14** with a high degree of stereocontrol (Scheme 3). [7] The *cis* stereochemistry observed in **11** might indicate a preference for a transition state where the bulky groups attain an equatorial conformation for minimal repulsions. Moreover, the reaction shows a high level of stereoselectivity in the formation of fused bicyclopentanols. Cyclization seems to proceed through a classical S_E_ mechanism involving stabilized carbocations β to silicon (the so-called β-effect). A unique feature of the reaction is the invariable formation of an exocyclic double bond by loss of the silicon group. The methylenecyclopentanol moiety is present in the skeleton of some naturally occurring terpene families.

Recent work has shown that the nature of the silyl group may cause important modifications in the mechanism pathway and therefore may change the final outcome. This is the case of analogous allylsilanes bearing the bulky t-butyldiphenylsilyl group, which give 3-cyclopenten-1-ols maintaining the hindered silyl group. [11–12]

The reaction between **2** and α,β-unsaturated acid chlorides provides an easy approach to silylated divinyl ketones **9–10** (Scheme 2), which are excellent precursors for silicon-directed Nazarov cyclizations. Acid catalysed electrocyclic closure (TFA, 0–20°C) allows the formation of exocyclic 2-methylenecyclopentan-1-ones **15–16** (Scheme 3), which are not easily prepared by classical methods, and for which few methods of synthesis have been reported in the literature. [7,13]

**Scheme 4:**
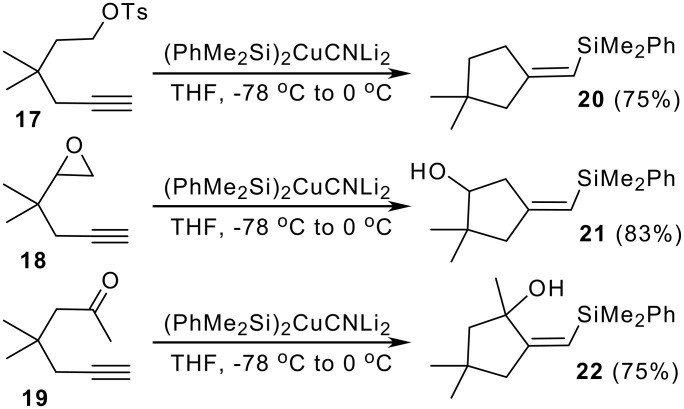
Silylcupration of terminal alkynes bearing electron-withdrawing functions.

Silylcupration of acetylenes is also a powerful tool for cyclopentane annulations. Terminal alkynes **17–19** bearing electron-withdrawing groups in appropriate positions undergo silylcupration-ring formation, when treated with higher order cyanocuprates as (PhMe_2_Si)_2_CuCNLi_2_. Intramolecular trapping of the vinylcuprate intermediate allows the synthesis of methylenecyclopentanes **20–22** (Scheme 4). [14]

**Scheme 5:**
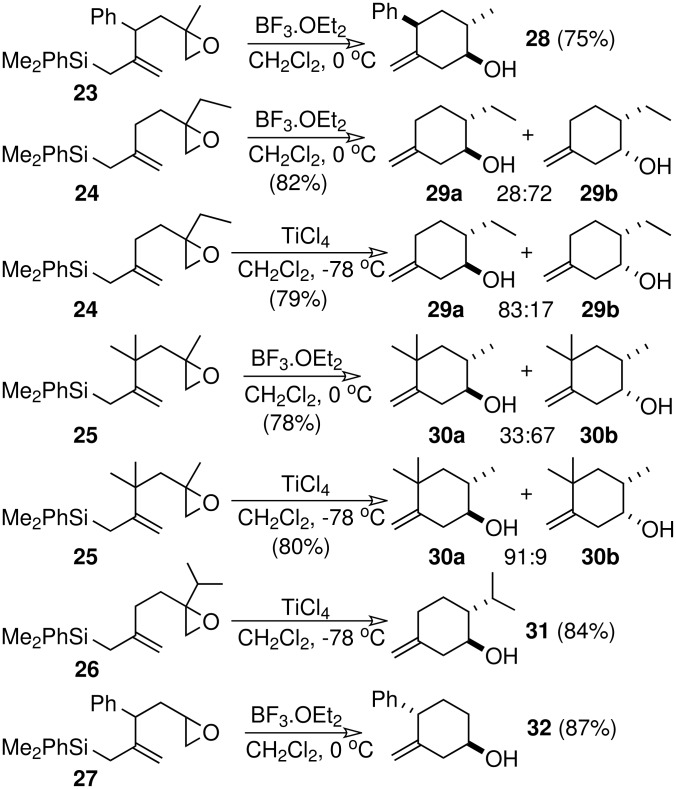
The acid-catalyzed cyclization of epoxyallylsilanes.

Epoxidation of the oxoallylsilanes obtained from the "silylcuprate methodology" provides a rapid access to epoxyallylsilanes. Thus, capture of intermediate **2** with enones and later treatment with sulfur ylides afford the epoxyallylsilanes **23–27** (Scheme 5). Despite its synthetic potential, the cyclization of epoxyallylsilanes has not been widely reported. Although Baldwin's rules predict that 5-*exo* attack, leading to cyclopentanols, is favoured over 6-*endo* attack, none of the former cyclization mode is observed when epoxyallylsilanes **23–27** are submitted to Lewis acidic conditions. Instead of this, a rearrangement-cyclization process, giving rise to 3-methylenecyclohexan-1-ols **28–32**, is observed when reaction is carried out in the presence of BF_3_ or TiCl_4_ (Scheme 5). [15]

The diastereoselectivity of the reaction depends on the Lewis acid used. Boron and aluminum-based catalysts show a preference for the *cis* isomer (**29b** and **30b**) whereas TiCl_4_ gives almost exclusively *trans* isomers (**29a** and **30a**). According to Schlosser, the preference for the *cis* isomer, when BF_3_ is used, might be due to the countercurrent flow of electrons in the Csp^2^-C(Si) and C = O bonds, which is favoured when these structural elements are aligned parallel. [16]

**Scheme 6:**
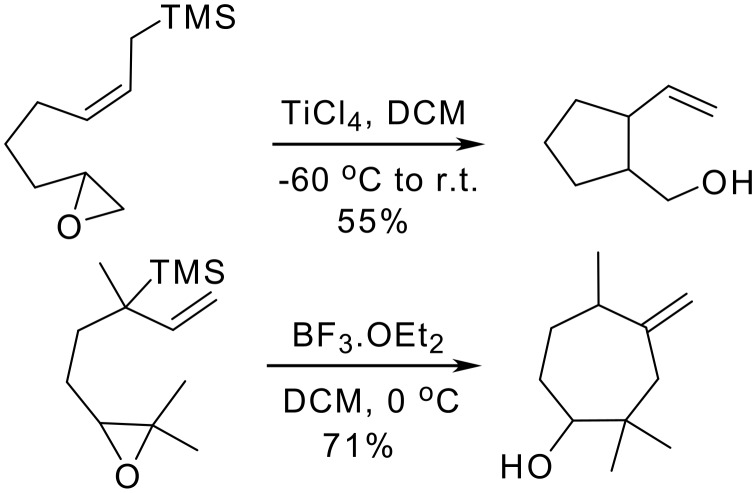
Intramolecular cyclization of TMS-epoxyallylsilanes.

Cyclization of epoxyallylsilanes containing the bulky t-butyldiphenylsilyl group takes place without loss of silicon giving cyclohexenols bearing the t-butyldiphenylsilyl group. [17] By contrast, the behaviour reported in the bibliography for trimethylsilylepoxyallylsilanes is frequently different from that observed for phenyldimethylsilylepoxyallylsilanes of type **23**, giving nucleophilic substitution at the most substituted carbon of the epoxide (Scheme 6). [18–19]

## Three and Four Membered Carbocycles

**Scheme 7:**
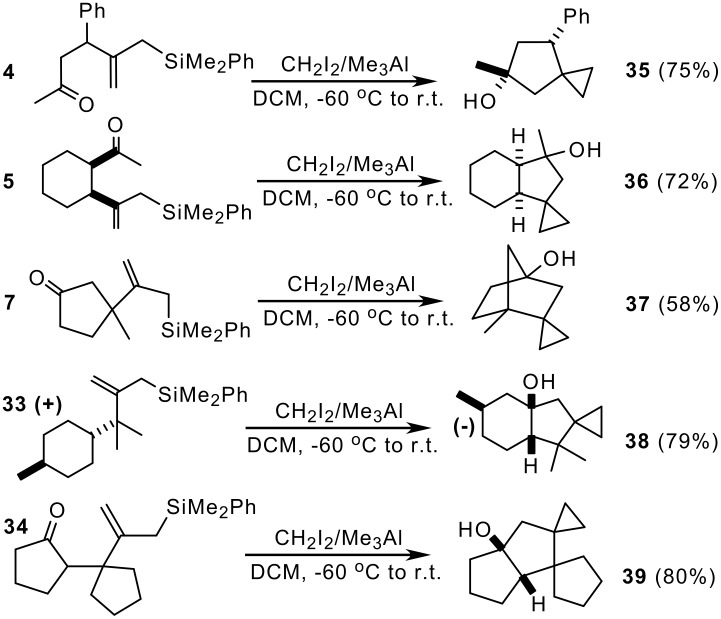
Spiro-cyclopropanation from oxoallylsilanes.

Oxoallylsilanes **4–7, 33** and **34**, readily available *via* silylcuprate addition of **2** to enones, react with CH_2_I_2_/Me_3_ Al at low temperature (-60°C to room temperature, then 48 h at r.t.) giving spiro-cyclopropanes **35–39** containing the spiro[2,4]heptanol moiety (Scheme 7). [20] High levels of stereoselectivity were found in all the examples studied. Formation of the spirocycle proceeds by a two-step pathway involving firstly, Me_3_Al-catalysed intramolecular cyclization of the oxoallylsilane and subsequent formation of a methylenecyclopentanolate, and then cyclopropanation. This unique mechanism enables the construction of hydroxylated bi-tri- and tetracyclic skeletons, bearing the spiro-cyclopropane moiety, from open chain allylsilanes in just one step. The high stereocontrol associated to the ring formation allows the synthesis of enantiomerically pure spiro-tricyclic alcohols containing an angular OH-group, such as **38** (Scheme 7). [20]

The use of reagents different from organoaluminun compounds resulted in poor efficiency and low stereoselectivity. For example, the Simmons-Smith reagent or the Furukawa modification (Et_2_Zn/CH_2_I_2_) is much less effective than the reported procedure. [21]

Unfortunately, this route cannot be used to synthesize spiro[2,5]octanes from epoxyallylsilanes of the type **23**, due to the high reactivity of the epoxide group towards Me_3_Al, the latter giving S_N_1 attack resulting in the formation of methyl alcohols to a great extent. Future work will show if cyclopropanating reagents with a weaker Lewis acid character can be appropriate to direct the reaction toward the synthesis of spiro[2,5]octanes, an structural moiety of interest in the synthesis of natural products.

**Scheme 8:**
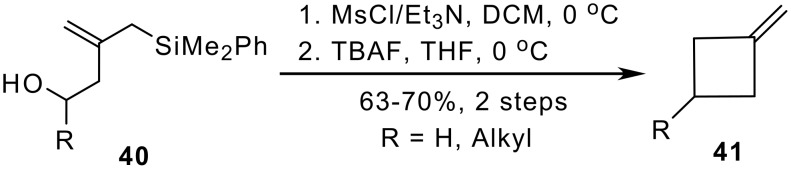
Cyclobutane formation from hydroxy-functionalized allysilanes.

Alcohols as **40** containing an allylsilane unit, which can be readily obtained by reaction of epoxides with the silylcuprate **2**, are excellent synthons for cyclobutane ring-formation. Formation of the corresponding mesylate and fluoride-induced intramolecular displacement led to methylenecyclobutanes **41** in good yields (Scheme 8). [22]

**Scheme 9:**
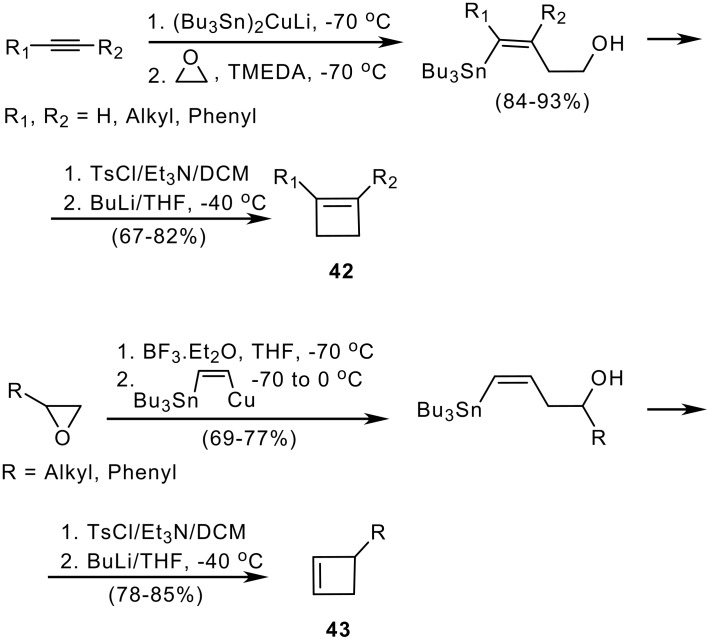
Cyclobutene formation from vinyltin cuprates and epoxides.

A different approach, starting from acetylenes instead of allenes and using silyl- or stannylcuprates followed by addition of an epoxide as electrophile, led to substituted cyclobutenes after cyclization of the vinylsilane or vinylstannane intermediate. [23] Cyclization of the corresponding vinylsilanes gave poor results of no synthetic utility, however the vinylstannane strategy results in formation of 1- and 3-substituted cyclobutenes **42** and **43** in good yield (Scheme 9).

As shown in Scheme 9, the strategy employed allows the selective formation or 1- or 3-substituted derivatives, where the coupling of a C_2_ acetylenic synthon and a C_2_ epoxide synthon provides a new and useful [2+2] annulation strategy for the preparation of the strained cyclobutene ring. The key step is the *syn* addition of the tin cuprate to the acetylene, which controls the *cis* stereochemistry required for cyclization. [23]

## Seven Membered Carbocycles

**Scheme 10:**
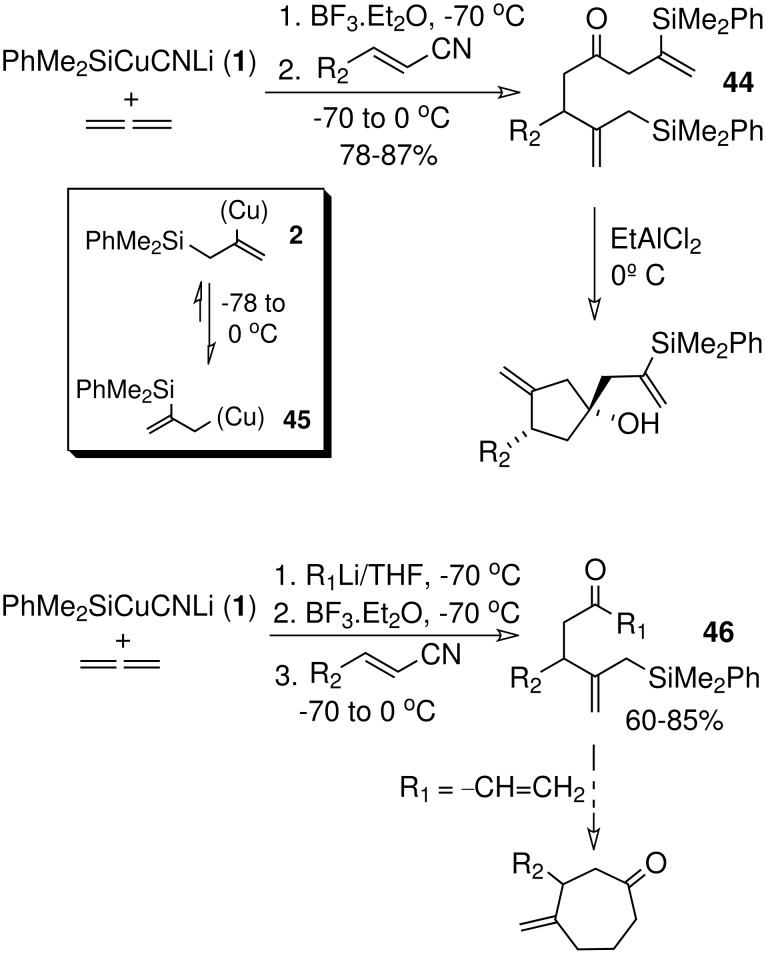
Silylcupration of 1,2-propadiene and reaction with α,β-unsaturated nitriles.

The use of nitriles and imines as electrophiles in the silylcupration of allene provides new alternatives for carbocyclization. Recently, we showed that α,β-unsaturated nitriles undergo a double addition process when treated with the cuprate species resulting from addition of **1** to allene, giving ketones **44** containing both an allylsilane group and a vinylsilane moiety (Scheme 10). [24] Equilibration between species **2** and **45** as the temperature rises from -70°C to 0°C must be the explanation for this surprising result. Whatever is the reason, this tandem process allows the introduction of two silylated functions, which display a markedly different reactivity. Effectively, allylsilane terminated cyclization, in the same conditions as before (see Scheme 3), gives chemoselectively methylenecyclopentanols, while the vinylsilane unit remains unchanged (Scheme 10). [24] Recent work revealed that addition one equivalent of organolithium reagent (R_1_Li) to the reaction mixture leads to the formation of ketones of type **46** (Scheme 10), which result from the addition of the two organometallic species present in the solution (silylcuprate and R_1_Li). When R_1_Li is an alkenyllithium this reaction opens new alternatives for preparation of 7-membered rings by intramolecular Michael addition of the allylsilane group to the enone (Scheme 10).

**Scheme 11:**
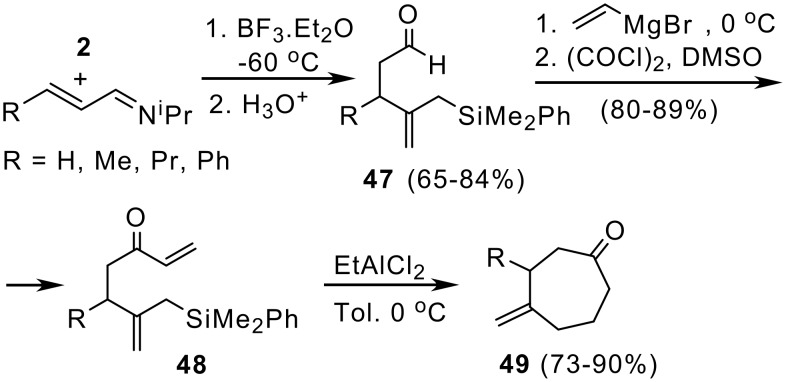
Cycloheptane formation from silylcupration of α,β-unsaturated imines.

Similarly, silylcupration of imines [25] provides a simple and efficient route for the preparation of seven membered carbocycles with different substitution patterns. Thus, reaction of **2** with α,β-unsaturated imines, at low temperature, affords allylsilane-containing aldehydes **47**, which upon addition of vinylmagnesium bromide followed by Swern oxidation lead to enones **48.** Lewis acid catalysed cyclization of **48** gives methylenecycloheptanones **49** in high yield (Scheme 11). [25] Consequently, oxoallylsilanes **47** can be considered as useful precursors for cycloheptane annulation. Moreover, the presence of an exocyclic double bond joined to the cycloheptanone core is a structural feature very common in many naturally occurring terpenes (Scheme 11).

**Scheme 12:**
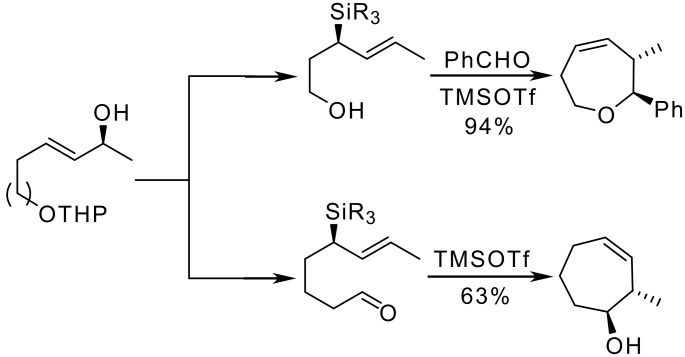
Seven membered ring formation from functionalized allylsilanes.

Other allylsilane-based strategies have been recently developed to build up cycloheptane derivatives. Thus, the synthesis of seven membered hydroxycycloalkenes and oxacycloalkenes has been achieved by intramolecular cyclization of functionalised allylsilanes obtained from optically active allylic alcohols (Scheme 12). [26]

## Conclusion

In summary, the metallocupration (Si-Cu and Sn-Cu) of allenes and acetylenes has proven to be extremely useful for the construction of cyclic structures ranging from three to seven membered rings, through processes which imply addition of the intermediate silylcuprate to an electrophile (enone, epoxide, nitrile, imine, etc) followed by Lewis-acid catalysed intramolecular cyclization, where the electrophile used determines the type of process and the size of the ring.
